# Chorion tissue- and plasma-derived extracellular vesicles exhibit superior anti-inflammatory and chondroprotective effects

**DOI:** 10.1186/s13287-025-04542-9

**Published:** 2025-08-05

**Authors:** Livia K. Fecskeova, Jana Matejova, Lucia Slovinska, Jana Bzdilova, Zuzana Kozovská, Denisa Harvanova

**Affiliations:** 1 Associated Tissue Bank, Faculty of Medicine, P. J. Safarik University and L. Pasteur University Hospital in Kosice, Tr. SNP 1, Kosice, 04011 Slovakia; 2 Cancer Research Institute, Biomedical Research Center SAS, Dúbravská cesta 9, Bratislava, 845 05 Slovakia

**Keywords:** Extracellular vesicles, Chorion tissue, Platelet-poor plasma, Tissue-derived EVs, MSC-EVs, Chondrocyte, Cell-free therapy

## Abstract

**Background:**

Extracellular vesicles (EVs) are the foundation of modern regenerative medicine using a cell-free approach. While current research mainly explores EVs from biological fluids and cell culture supernatants, tissue-derived EVs hold great promise, but remain largely underexplored. Since healthy placental tissues such as the chorion are widely available after full-term delivery, ethically unobjectionable, and possess exceptional regenerative potential, we sought to compare the biological effects of EVs derived directly from chorion tissue with those from chorion-derived mesenchymal stromal cell EVs and plasma EVs.

**Method:**

We compared the biological impact of EVs from various sources (chorion tissue CHO-Ti, MSCs from chorion CHO-MSC and platelet-poor plasma PPP) and isolated by various techniques on the gene expression of osteoarthritic chondrocytes. Additionally, we assessed the effect of enriched soluble proteins of CHO-MSC and CHO-Ti secretome vs. their EVs. EVs were characterized by particle number and size (NTA), protein content (BCA assay) and immunophenotype (flow cytometry). Changes in gene expression of chondrocytes were quantified by RT-qPCR.

**Results:**

CHO-Ti-EVs and PPP-EVs showed particularly beneficial effect on the inflammatory process, with their biological impact surpassing that of CHO-MSC-EVs. Chondroprotective markers *COL2A* and *ACAN* were robustly upregulated by CHO-Ti-EVs and PPP-EVs but showed only modest or variable increases with CHO-MSC-EVs. *COMP* expression, however, was specifically enhanced by CHO-MSC-derived components. Furthermore, our results also indicate that the therapeutic properties of the CHO-Ti secretome are exclusively linked to EVs. Among CHO-MSC-EVs, purification combined with UC resulted in the highest purity, however EVs purified by SEC presented a more favourable surface marker profile and better biological effects. The observed variability suggests that different EV preparations harbour distinct subpopulations that influence regulatory pathways differently and highlight the importance of EV source and isolation methodology in determining biological activity.

**Conclusion:**

CHO-Ti-EVs showed promising effects on cartilage regeneration and inflammation modulation, suggesting they may represent a viable alternative to plasma- and CHO-MSC-EVs. Moreover, the chorion represents a readily accessible and abundant source of perinatal tissue obtainable non-invasively after full-term delivery, further supporting the translational potential of CHO-Ti-EVs.

**Supplementary Information:**

The online version contains supplementary material available at 10.1186/s13287-025-04542-9.

## Background

While cell therapy based on mesenchymal stromal cells (MSCs) was traditionally favoured for their multi-differentiation potential and immunosuppressive properties [1], the past decade has seen a decisive shift toward harnessing the therapeutic potential of their secretome, particularly via extracellular vesicles (EVs) [2]. EVs play a pivotal role in intercellular communication and hold great promise as multifaceted mediators in diagnostics, therapeutics, and molecular cargo delivery. The application of EVs derived from MSCs has become a key approach of cell-free regenerative medicine, independent of MSC tissue source.

Among degenerative joint disorders, osteoarthritis (OA) stands out as the most prevalent. It is a chronic, painful, whole-joint disease characterized by structural alterations in the articular cartilage, subchondral bone, synovial membrane (synovitis), meniscal degeneration and hypertrophy of the joint capsule [3]. The progression of OA is driven by immune system activation and an imbalance between anabolic and catabolic processes, ultimately resulting in cartilage degradation, bone remodelling and joint dysfunction [4]. Multiple studies confirmed that MSC-EVs play a therapeutic role in the pathological process of cartilage injury, by their ability to modulate inflammatory response and stimulate OA chondrocytes to produce extracellular matrix (ECM) [5–9].

In addition to MSC-derived EVs, it is important to consider other sources of EVs that may offer complementary or even superior therapeutic benefits. EVs are secreted by all cell types and circulate in all body fluids, with blood plasma being a particularly rich source, making plasma-derived EVs (PL-EVs) a key focus for their diagnostic value in various diseases [10], including OA [11]. Given the long-standing use of blood products like platelet-rich plasma (PRP) in OA therapy, PL-EVs bear strong potential for facilitating regenerative processes. Preliminary studies show that PL-EVs can exert regenerative effects by modulating inflammation [12, 13], increasing the expression of ECM-building genes [13–16], promoting proliferation [17, 18] and tissue repair [12], and polarizing macrophages [19]. Due to advantages such as ease of collection, lower immunogenicity, and the potential for large-scale production, PL-EVs are already on the rise as a promising alternative to MSC-EVs [12, 20]. While some studies support regenerative potential of total PL-EVs, others emphasize the need to isolate EVs from specific plasma fractions to minimize the influence of cellular contaminants. For this reason, in this study the use of platelet-poor plasma (PPP) was selected, which minimizes the presence of platelets, platelet-derived vesicles, and other cellular components such as erythrocytes and leukocytes abundant in platelet-rich plasma (PRP) and could confound downstream analyses. The use of PPP thus improves the specificity and quality of EVs preparation.

Based on the source, from which they are obtained, EVs can be generally classified into three subtypes: cell culture-derived EVs (such as MSC-EVs), body fluid-derived EVs (like PPP-/PRP-EVs), and tissue-derived EVs (Ti-EVs) [21]. Current research mainly focuses on EVs present in biological fluids and cell culture supernatant, yet the studies of Ti-EVs are becoming increasingly attractive, with significant potential for both diagnostic and therapeutic applications [21]. Compared to EVs derived from cell cultures and body fluids, Ti-EVs retain more of their original information and more accurately reflect intercellular communication [22].

However, due to limited tissue sources, tissue heterogeneity or difficult preservations, MSC-EVs are still the preferred choice for therapeutic usage. Placental tissues, on the other hand, are abundantly available and ethically unobjectionable and, because they are discarded post-partum, they can be widely used for extensive research and therapeutic studies [23]. MSCs of neonatal origins, such as chorion- and amnion-derived MSCs, exhibit superior proliferation ability, high immunosuppressive capacity, robust angiogenic potential [24], delayed onset of senescence [25], high level of VEGF factor secretion [26] and lower immunogenicity when compared with MSCs classically derived from the adult bone marrow [27]. Moreover, amnion and chorion are tissues of early embryologic origin that may entail progenitor potential. The membrane is embedded with a wide array of growth factors (TGF-β, FGF, PDGF, EGF, VEGF, PIGF), which play essential roles in promoting cellular proliferation and differentiation, and collagen, which aids tissue healing, regeneration and repair [28]. Since EVs reflect the properties of their parental cells, chorion- and amnion-derived products may offer similar benefits *via* a cell-free approach.

Apart from the source of the EVs, isolation technique represents another critical factor that may introduce considerable variations into their biological effects. Currently, based on published literature, the three most used EV isolation methods are ultracentrifugation (41.5%), size exclusion chromatography (SEC) (20.1%) and precipitation with polymers (11.3%) [29]. Selecting an appropriate EVs isolation method is crucial, as it directly impacts the purity, yield, and functional integrity of EVs, reflecting in their therapeutic application and biological activity. However, each method differs in principle, efficiency, and purity, with unique advantages and limitations that must be carefully considered based on the specific research or clinical context.

Building upon these promising indications, the aim of this study was to evaluate and compare the regenerative potential of EVs from chorion tissue (CHO-Ti) alongside the already acknowledged PPP- and CHO-MSC-EVs, in the context of OA chondrocytes in vitro. Additionally, we compared CHO-MSC-EVs isolated by different protocols to assess the impact of the isolation method on their regenerative efficacy.

Chondrocytes were chosen as the target cell type due to their exclusive presence in articular cartilage and their key role in synthesizing and maintaining ECM, making them a relevant model for studying regenerative processes. Since ECM homeostasis is central to cartilage repair, assessing the effects of EVs on chondrocytes offer direct insight into their therapeutic potential.

## Materials and methods

**Fig. 1:**
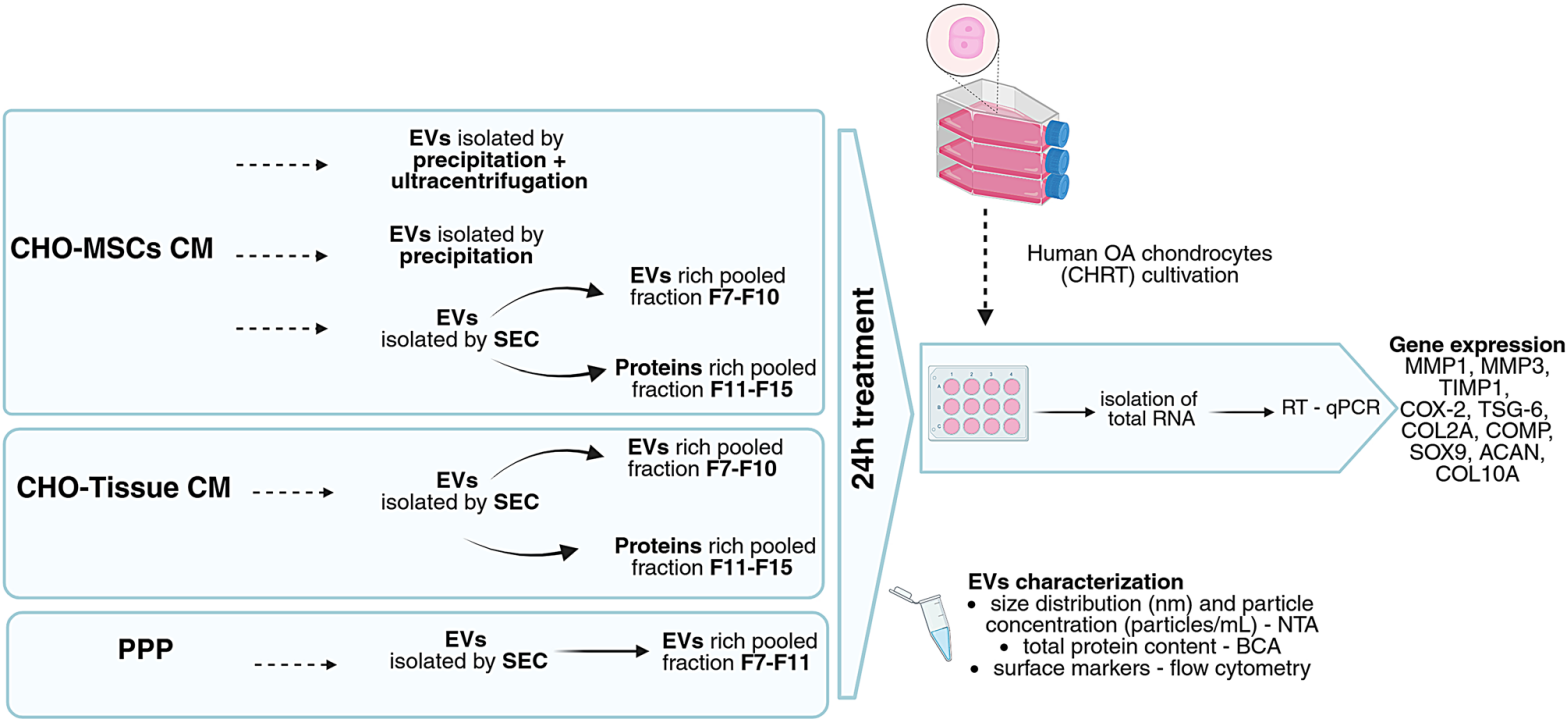
Workflow of the experiments. Isolation and characterization of EVs/proteins of various sources and isolated by various methods, and their application to chondrocytes

Experimental design is depicted on Fig. 1.

### Collection of biological samples

The study was approved by the Ethics Committee of the P. J. Safarik University and L. Pasteur University Hospital in Kosice, Slovakia (Approval ID 2023/EK/06026 and 2023/EK/06028) and was conducted in accordance with the declaration of Helsinki. All donors gave their informed consent for inclusion before they participated in the study.

Chorionic tissue (*n* = 4) was obtained from healthy donor mothers less than 30 years old. Whole blood samples (*n* = 4) were obtained from healthy donors, without previous fasting. Cartilage tissue (*n* = 4) was obtained from OA patients with Kellgren–Lawrence grade III/IV undergoing total knee replacement.

Overview of donor characteristics and eligibility criteria for each tissue source are summarized in the Supplementary material (Suppl. Tab. S1).

### Chorionic tissue, isolation of MSCs and collection of conditioned media

Fetal membranes were processed on the same day as the caesarean section. Immediately, after collection as a biological waste, the tissue was transferred into the transport medium (DMEM, Sigma Aldrich, Germany) supplemented with 1% (v/v) antibiotic/antimycotic solution containing penicillin (100 U/mL), streptomycin (100 µg/mL) and amphotericin (0.25 µg/mL) (Sigma Aldrich, Germany) and transported. All samples were processed within 2 to 3 h post-delivery to preserve tissue integrity and cellular viability. Part of the chorion (10 × 10 cm) was dissected manually and washed intensively in PBS containing 1% (v/v) antibiotic/antimycotic solution to remove red blood cells. After processing, the tissue was weighed, and DMEM was added at 150 mg tissue/mL. The tissue sample was incubated in DMEM for 24 h. Conditioned medium (CM) (CHO-Ti-CM) was collected, centrifuged at 300 ×g for 10 min at 4°C to remove cell debris, filtered through a 0.22 μm filter and stored at − 80 °C until use. CHO-Ti-CM was used for the isolation of EVs (CHO-Ti-EVs). MSCs were isolated from CHO-Ti. Chorion was cut into small pieces (1.5 × 1.5 cm) and treated with 0.1% (v/v) bacterial collagenase type II (Gibco, USA) for 4 h at 37 °C. After digestion, the pieces of tissues were filtered through a 40 μm cell strainer (Sarstedt AG & Co., Germany) and the filtrate was centrifuged at 300 ×g for 7 min. The obtained chorionic cells were cultured in α-MEM, supplemented with 10% FBS, 1% (*v*/*v*) antibiotic/antimycotic solution and 1% L-glutamine (all Sigma Aldrich, Germany). Cells were seeded and maintained in 75 cm^2^ culture flasks (Sarstedt AG & Co., Germany) at the density 5 × 10^4^/cm^2^. Non-attached cells were removed after three to five days of incubation. The cultivation medium was changed twice a week.

When CHO-MSCs reached 80% confluence, cells were carefully washed in PBS, medium was changed to DMEM for 24 h. CM (CHO-MSC-CM) was collected after and centrifuged at 300 ×g for 7 min at 4 °C, filtered through 0.22 μm filter, concentrated 5× using Amicon^®^ Stirred Cells with Ultracel^®^ 100 kDa Ultrafiltration Discs (Merck Life Science, USA) and stored at − 80 °C until use. CHO-MSC-CM was used for the isolation of EVs (CHO-MSC-EVs).

All incubations were done at 37 °C, 95% humidity and in a 5% CO_2_ atmosphere.

Immunophenotypic characterization and trilineage differentiation of CHO-MSCs can be found in the Supplementary material.

### Isolation of CHO-EVs

#### Isolation of CHO-MSC-EVs by precipitation (P) and ultracentrifugation (UC)

Precipitation (CHO-MSC-EVs-P): CM was mixed with 10% PEG 8000 and incubated overnight at 4 °C. After incubation, the mixture was centrifuged at 1500 ×g for 30 min and pellet was resuspended in sterile PBS and stored at − 80 °C.

Precipitation + ultracentrifugation (CHO-MSC-EVs-UC): EVs precipitation by PEG was followed by ultracentrifugation at 100.000 ×g for 1 h. Supernatant was discarded and EVs were diluted with sterile PBS and stored at − 80 °C.

#### Isolation of CHO-MSC-EVs and CHO-Ti-EVs by SEC

Fractions of EVs and soluble proteins were separated from CM by SEC using Gen 2 qEV original ~ 35 nm columns at RT. First, CHO-MSCs-CM or CHO-Ti-CM was concentrated to 500 µl using Amicon Ultra-15 centrifugal filter unit with 3 kDa cut-off membrane (Merck Life Science, USA). Such concentrated CM was directly loaded onto the loading frit of SEC column and fractions were collected immediately in 500 µL volumes. In total, 15 fractions were eluted in PBS during one separation process and collected as F1–F15. Fractions F7–F10, rich in EVs, were pooled and represented the EVs sample (CHO-Ti-EVs or CHO-MSC-EVs-SEC); fractions F11-F15, rich in proteins, were also pooled and represented the proteins sample (CHO-Ti-PR or CHO-MSC-PR). EVs and proteins samples were further 2× concentrated at 14.000 ×g for 5 min using Amicon Ultra 100 K centrifugal filter unit. Aliquots were stored at − 80 °C until subsequent analyses.

### Preparation of platelet-poor plasma (PPP) and isolation of EVs by SEC

10 mL of whole blood from each donor (*n* = 4) was collected into citrate tubes (Sarstedt AG & Co, Germany) and processed within 30 min of sampling. The platelet-poor plasma (PPP) was prepared in 3 centrifugation steps as it was described previously [30]. After the last centrifugation step the upper part consisted of PPP and the lower part of PRP. PPP was collected, pooled, aliquoted and used for EVs isolation by SEC using qEVoriginal ~ 35 nm columns.

PPP samples were centrifuged at 10.000 ×g for 10 min and were directly loaded onto the loading frit of SEC columns. PPP-EVs were separated and collected into 500 µL aliquots, as written above for CHO-MSC-CM. Fractions rich in EVs, F7–F11 were pooled and concentrated 2× at 14.000 ×g for 5 min using Amicon Ultra 100 K centrifugal filter unit (Merck Life Science, USA). Aliquots of PPP-EVs were then stored at − 80 °C until subsequent analysis.

### EVs characterization

#### Total protein content

The total protein concentrations from all samples (EVs and proteins fractions) were evaluated by a Pierce™ Rapid Gold bicinchoninic acid (BCA) assay kit (Thermo Scientific, USA), in accordance with the manufacturer’s recommendations, and analysed on a multimode reader (TRISTAR, Berthold Technologies). Relative purity of the sample was calculated as the ratio of particle number/mL to protein concentration in mg/mL, divided by 10^11^; the resulting number serves to compare samples to each other, with higher number corresponding to higher purity.

#### Nanoparticle tracking analysis

NanoSight NS500 system (Malvern Instruments Ltd., UK) equipped with a 405 nm laser and an sCMOS trigger camera (set on level 12) was used for analysis of the concentration, particle size, and their distribution. Samples were diluted with DPBS w/o Ca and Mg (PAN Biotech, GmbH, Germany) or in ultrapure water before measurement to obtain a particle concentration between 1 and 10 × 10^8^/mL suitable for NTA analysis. 3–5 videos with duration 30 s were recorded for each sample. The video analysis was done with the NanoSight Software NTA v2.3. The detection threshold was set on 10.

#### MACSPlex exosome assay

MACSPlex Exosome Kit (Miltenyi Biotec, Germany) was used for the analysis of EVs’ 37 surface APC-conjugated epitopes and two isotype controls according to the manufacturer’s instruction and as described previously [11]. Signal intensity in each 39 specific bead populations were measured on a Becton Dickinson FACSCalibur using CellQuestPro software (Becton Dickinson, Belgium). MFI measured within each gate of an individual bead population was normalized against the mean MFI values of tetraspanins CD9/CD63/CD81 in order to determine the relative levels of a surface marker.

### Collection of cartilage and isolation of chondrocytes

Chondrocytes were isolated from human OA cartilage by enzymatic digestion with 1.0 mg/mL collagenase type II (Gibco, USA) in DMEM containing 1% (v/v) antibiotic/antimycotic solution overnight at 37 °C under continuous rolling. The digested fragments were then filtered through a 70 μm cell strainer (Sarstedt AG & Co., Germany). The cell suspension was centrifuged at 300 ×g for 10 min at 4 °C, washed twice in DMEM. Cells were cultivated in the normal cultivation medium consisting of DMEM/Nutrient Mixture F-12 Ham (Sigma Aldrich, Germany) supplemented with 1% (*v*/*v*) antibiotic/antimycotic solution, 10% FBS, 1% Insulin-Transferrin-Selenium (100×, Gibco, USA) at 37 °C, 95% humidity and 5% CO_2_.

### Immunofluorescence characterization of chondrocytes

Cells were fixed with 4% paraformaldehyde for 20 min, washed with 0.1 M PBS, blocked with 10% normal gout serum (NGS) or 10% normal donkey serum (NDS) for 1 h at RT and then incubated overnight at 4 °C with one of the following primary antibodies: mouse aggrecan antibody (1:100, Abcam), rabbit collagen II antibody (1:200, Abcam) and Pro Collagen I alpha 1 antibody (1:20, R&D Systems). Subsequently, cell cultures were washed with 0.1 M PBS and incubated with species-appropriate fluorescent secondary antibodies (anti-mouse, anti-rabbit, or anti-sheep antibodies, Alexa Fluor 594, 488, 1:200, Abcam) for 1 h at RT and by DAPI (Sigma-Aldrich). Immunofluorescence positivity quantification was performed on a CytellCell Imaging System (GE Healthcare, Life Science) and ImageJ software, within 10 different fields (500 × 500 μm) for each marker.

### Chondrocytes treatment

When chondrocytes reached 80% confluence, they were passaged and seeded on a 24-well plate in density 50.000 cells/well and cultured for 7 days under normal conditions. After 7 days, the normal cultivation medium was changed to medium with Exosome-Depleted FBS (Gibco™) and individual treatments were started: enriched soluble protein fractions CHO-Ti-PR and CHO-MSC-PR were added in concentration 0.65 mg/mL and 1.60 mg/mL, respectively, and all the isolated EVs (PPP-EVs, CHO-Ti-EVs, CHO-MSC-EVs-P, CHO-MSC-EVs-UC, CHO-MSC-EVs-SEC) were added in concentration 1 × 10^9^ EVs/50.000 cells (or 1 × 10^9^ EVs/mL medium), based on previous studies [13, 16, 31, 32]. Chondrocytes with different treatments were incubated for 24 h, comparably to previous studies [32, 33], then the media were discarded and 500 µL TRIzol reagent (Invitrogen™) was added to each well for cell lysis. Lysates were collected and stored at -80 °C till RNA extraction. Experiment was repeated with 4 biological replicates of chondrocytes.

### Gene expression analysis by RT-qPCR

Total RNA was isolated from approximately 1 × 10^5^ chondrocytes using TRIzol reagent (Invitrogen™) and RNeasy Micro kit (Qiagen) according to the manufacturer’s protocol. The quality and quantity of RNA samples were inspected on NanoDrop spectrophotometer. Accordingly, 200 ng of RNA was reverse transcribed using oligo(d)T primers and the SuperScript™ VILO™ cDNA Synthesis Kit (Invitrogen™) following the manufacturer’s protocol. The obtained cDNA was diluted 10× and used directly in quantitative PCR (qPCR) or stored at − 80 °C. qPCR was performed using the PowerUp™ SYBR™ Green Master Mix (Applied Biosystems) on a CFX96 Real-Time Detection System (Bio-Rad). A 10 µL reaction contained 1× PowerUp Sybr Green master mix, 500 nM of each forward and reverse primer and 2 µL of 10× diluted cDNA. Relative gene expression of the target genes was normalized to the housekeeping genes *GAPDH* and *YWHAZ* and calculated as normalized relative expression. The log2 value of the fold change was used for visualizations and for statistics. Relative quantity (for chondrocytes phenotype) was calculated as 2^-ΔCt^. PCR conditions, the list of target and housekeeping genes and their primer sequences can be found in Supplementary Table S2.

### Sulphated glycosaminoglycan (sGAG) assay

Chondrocytes of the 1^st^ passage were seeded on a 24-well plate in density 50.000 cells/well and cultured for 7 days. After 7 days, cultivation medium was changed to medium with Exosome-Depleted FBS (Gibco™) and treatment with EVs isolated from CHO-MSCs, CHO-Ti and PPP started. Chondrocytes were cultivated with EVs for 10 days. Total cellular and extracellular content of sGAG was determined from the collected supernatant following papain extraction for 3 h at 65 °C, using the Blyscan Assay kit (Biocolor, UK), according to the manufacturer’s protocol. Amount of GAG (µg/mL) in individual samples was normalized to total protein content measured by BCA protein assay.

### Statistics

Statistical analysis was performed using GraphPad Prism version 8.0.0 for Windows, GraphPad Software (San Diego, CA, USA). Results of gene expression and sGAG production were analysed using one-way parametric ANOVA and Tukey’s post-hoc tests. EVs surface markers expressions were analysed by an unpaired t-test. *p* < 0.05 was considered significant. Data are presented either as individual replicates or as the mean ± SD.

A principal component analysis (PCA) was performed to reduce the dimensionality of the dataset of 37 EV surface markers of all EV groups, and to reveal the clustering of samples in each group. PCA was calculated in R (v4.1.1) on scaled variables using the packages FactoMineR [34], factoextra [35] and visualized by ggplot2 [36].

## Results

### Characterization of chondrocytes and MSCs

**Fig. 2:**
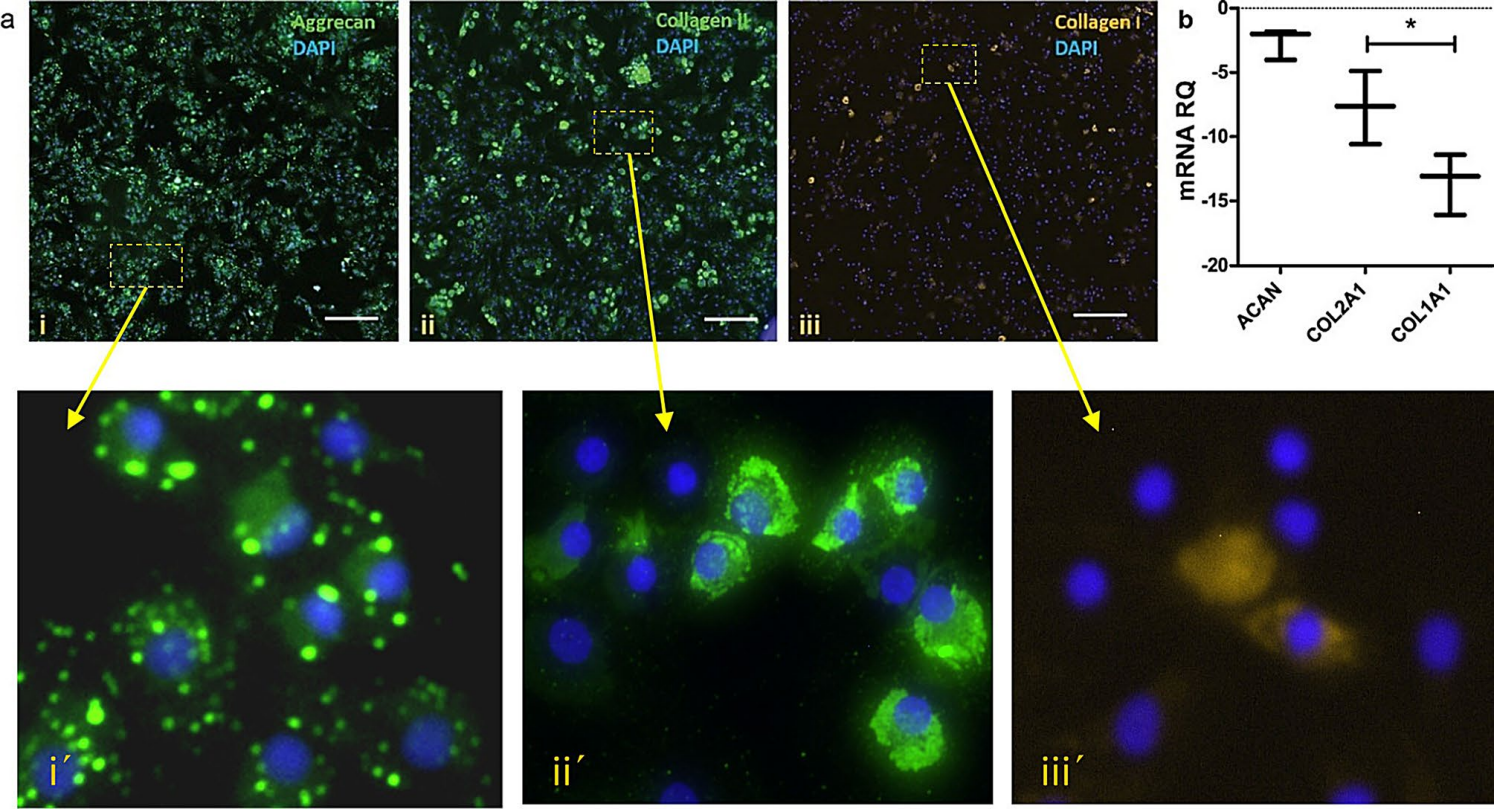
Analysis of chondrocyte’s phenotype (**a**) by immunofluorescence: The percentage of chondrocytes expressing aggrecan (i), collagen type II (ii), or collagen type I (iii) over the total chondrocytes was calculated using ImageJ software, within 10 different fields (500 × 500 μm) for each marker. Details of labeled cells (squares) show expression of aggrecan (i´), type II collagen (ii´) or type I collagen (iii). Scale bars (i, ii, iii) = 200 μm; (i´, ii´, iii´) = 50 μm; (**b**) by RT-qPCR shown as the log2-value of the relative quantity (RQ) of gene expression of collagen type I, type II and aggrecan (*ACAN*), normalized to *GAPDH*, in three replicates of chondrocytes. Statistical significance calculated by t-test. *p < 0.05, ** *p* < 0.01, *** *p* < 0.001

To confirm that chondrocytes retained their true phenotype (and did not differentiate into fibroblasts), cellular content of aggrecan, collagen type II vs. type I was quantified by RT-qPCR and by immunofluorescent staining. Immunofluorescent analysis revealed that 70.2 ± 1.2% of chondrocytes were positive for collagen type II, and 85.1 ± 2.3% for aggrecan, and only 12.7 ± 1.7% for collagen type I (Fig. 2a). Similar results were obtained from the gene expression analysis, that showed that chondrocytes highly expressed chondrogenic marker genes (collagen II and aggrecan), moreover aggrecan was expressed in amounts comparable to *GAPDH*, while collagen type I, which indicates the presence of fibrotic connective tissue, was expressed in significantly lower amounts compared to collagen type II (Fig. 2b). These results confirmed the true nature of chondrocytes used in our experiments.

The characterization of CHO-MSCs’ immunophenotype was performed by flow cytometry. Analysis indicated that CHO-MSCs were highly positive for MSC-specific surface markers (CD73, CD90, CD105; Suppl. Fig. S1). MSCs also demonstrated the capacity to differentiate into adipogenic, osteogenic, and chondrogenic lineages (Suppl. Fig. S2). These results confirm the multipotent nature and identity of the MSCs used in this study.

### EVs isolated from various biological sources and by various techniques

#### General characterization

**Fig. 3:**
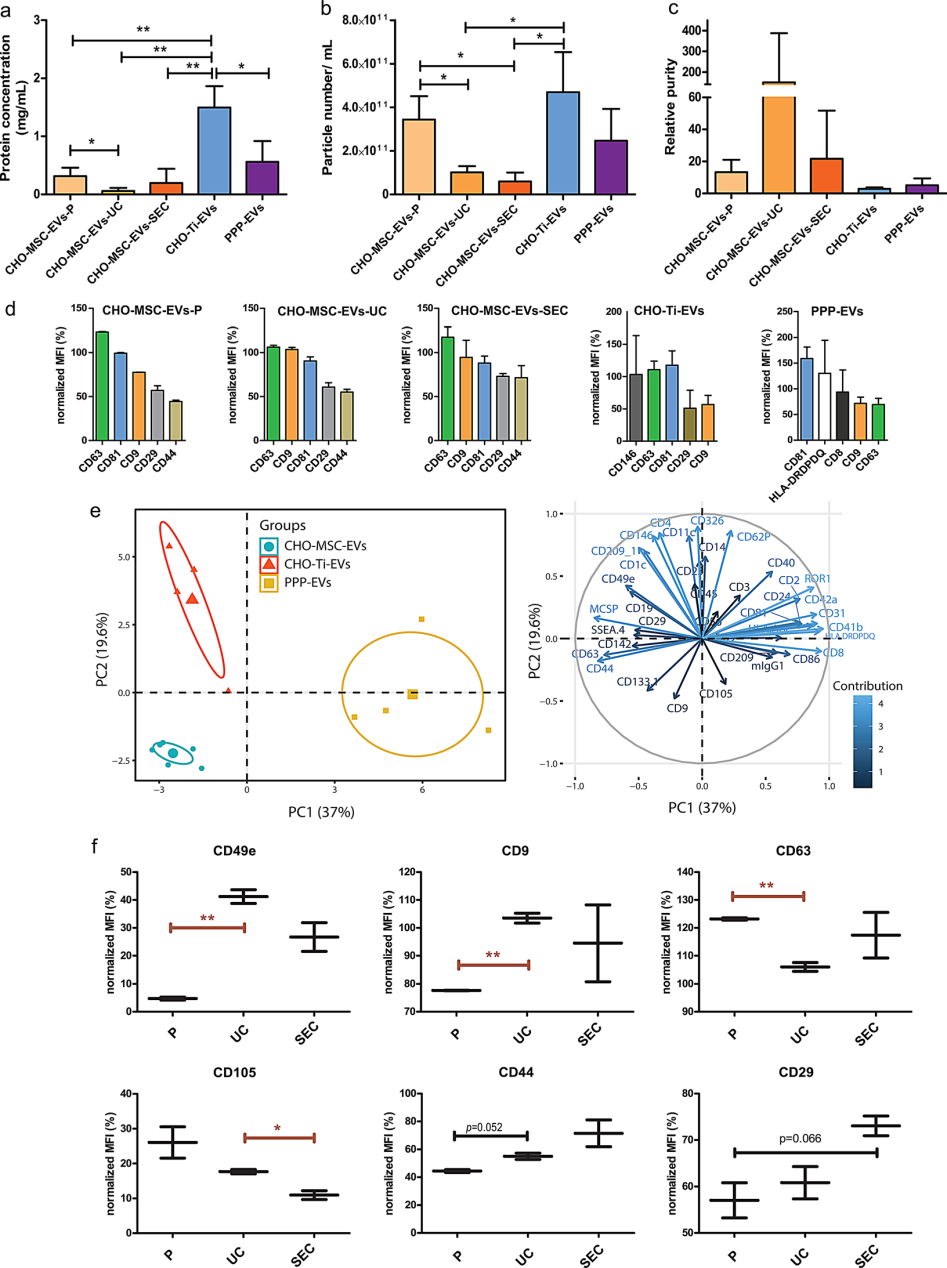
Characterization of EVs. (**a**) Protein concentrations by BCA, (**b**) particle number/mL by NTA and (**c**) relative purity of EV samples isolated by various techniques and from various sources. Mean of 3 biological replicates ± SD. *p < 0.05; **p < 0.01; ***p < 0.001. (**d**) Expression of the top 5 EV surface markers, including the EV-specific surface markers CD81, CD63 and CD9, by MACSPlex analysis. Data were normalized to mean fluorescence intensity (MFI) of CD9/CD63/CD81 of EVs. (**e**) PCA analysis of 37 EV surface markers of EVs isolated from various sources (PPP *n* = 4, CHO-MSCs *n* = 6, CHO-Ti *n* = 4). Ellipses represent the 95% confidence intervals with centred symbols representing the mean value. (**f**) Different expression of EV surface markers of CHO-MSC-EVs based on the isolation technique. Mean ± SD. *p < 0.05; **p < 0.01; ***p < 0.001

To compare EVs, three biological sources were used: PPP, CHO-Ti and CHO-MSC (CHO-MSC-EVs-SEC), all isolated by SEC. CHO-MSC-EVs were also isolated using two other methods: precipitation (CHO-MCS-EVs-P) and precipitation followed by ultracentrifugation (CHO-MSC-EVs-UC). Average particle sizes ranged from 120.20 ± 18.54 nm for PPP-EVs to 165.35 ± 35.0 nm for CHO-MSC-EVs-P (Table 1). Isolation by precipitation yielded the highest number of CHO-MSC-EVs, with a statistically significant difference between CHO-MSC-EVs-P and EVs isolated by the two other methods (vs. CHO-MSC-EVs-UC *p* = 0.0192, vs. CHO-MSC-EVs-SEC *p* = 0.0126) (Fig. 3a). CHO-MSC-EVs-UC contained the least contaminating proteins (Fig. 3b) and the highest sample relative purity (Fig. 3c). PPP- and CHO-Ti-EVs (both isolated by SEC) resulted in the highest particle count per mL but contained more contaminating proteins. The best yield and lowest protein contamination was achieved by the combination of precipitation and UC.

**Table 1:** Comparison of particle size, number of particles/ml and protein concentration in various EV preparations measured by NTA and BCA assay

Sample	Particle size ± SD (nm)	Particles/ mL ± SD (× 10^11^)	Protein concentration ± SD (mg/mL)
CHO-MSC-EVs-P	165.35 ± 35.0	3.44 ± 1.07	0.32 ± 0.15
CHO-MSC-EVs-UC	151.48 ± 20.84	1.02 ± 0.28	0.06 ± 0.05
CHO-MSC-EVs-SEC	152.13 ± 21.21	0.60 ± 0.41	0.20 ± 0.25
CHO-Ti-EVs	161.92 ± 18.46	4.69 ± 1.85	1.50 ± 0.37
PPP-EVs	120.20 ± 18.54	2.23 ± 1.87	0.56 ± 0.36

#### EVs surface markers

Analysis of surface markers expression revealed that EVs from different sources and isolation techniques were all highly positive for EV-specific surface markers CD81, CD63, and CD9, and these were the most expressed in all CHO-MSC-EVs, regardless of the isolation technique (Fig. 3d). CHO-MSC-EVs also expressed MSC-specific markers CD29 and CD44, while showing minimal levels of hematopoietic marker CD45 (2.78 ± 4.05) and HLA-DR (0.52 ± 0.15). Among all EV samples, PPP-EVs exhibited the highest CD81 expression but the lowest CD63. The expression of all surface markers of isolated EVs are summarized in the Supplementary material (Suppl. Fig. S3 and S4).

PCA analysis of 37 EV surface markers showed a clear distinction between EVs derived from PPP, CHO-Ti, and CHO-MSCs (Fig. 3e), due to the presence of source-specific markers. For example, PPP-EVs had substantially higher levels of HLA-DR, CD8, CD41b and CD42a compared to other EVs. CD146 was the most expressed marker on CHO-Ti-EVs. Even though CHO-MSC-EVs clustered closely together regardless of the isolation technique, differences in the expression of some markers were observed. The most differences were observed for CHO-MSC-EVs-P, such as significantly lower levels of CD49e and CD9, and higher levels of CD105 and CD63 in comparison with other methods (Fig. 3f). Conversely, marker CD105 had the lowest expression in CHO-MSC-EVs-SEC, such as other non-MSC related markers, like CD142, CD45, HLA-ABC (Suppl. Fig. S4), however CD146 and MSC-specific markers CD44 and CD29 were the highest in CHO-MSC-EVs-SEC (Fig. 3f).

### The biological effect of EVs and soluble proteins on OA chondrocytes

#### Comparison of EVs

**Fig. 4:**
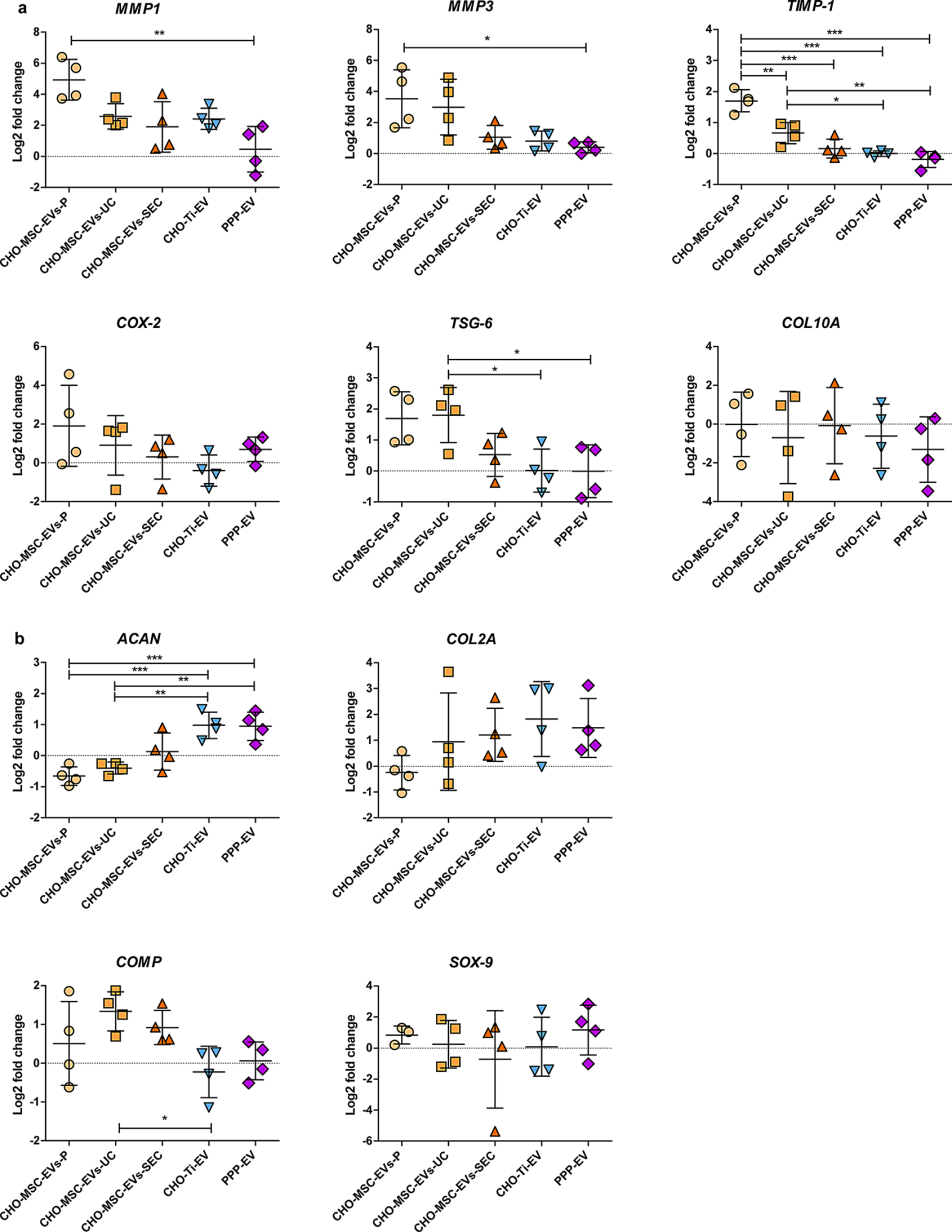
Changes in the expression of pro-inflammatory/degradation (**a**) and chondroprotective (**b**) genes of chondrocytes co-cultivated with EVs isolated by different methods (P precipitation, UC precipitation + ultracentrifugation, SEC size exclusion chromatography) and from various biological sources (CHO-MSCs, CHO-Ti, PPP). Expression is shown as the log2 value of fold change, compared to expression of the untreated control. Symbols represent individual replicates, the mean ± SD is shown. *p < 0.05; **p < 0.01; ***p < 0.001

The effect of a 24 h co-cultivation of a single dose of EVs with OA-derived chondrocytes was evaluated compared to untreated chondrocytes using gene expression analyses. CHO-MSC-EVs-P consistently had the most negative impact, significantly increasing the expression of inflammatory (*COX-2*, *TSG-6*) and degradation (*MMPs*, *TIMP-1*) genes across all replicates (Fig. 4a). CHO-MSC-EVs-UC performed slightly better, as the additional ultracentrifugation step improved the quality of EVs. Among CHO-MSC-EVs, those isolated via SEC yielded the best results. However, CHO-Ti-EVs and PPP-EVs outperformed all CHO-MSC-EVs, demonstrating the lowest expression of key inflammatory mediators.

In the expression of the chondroprotective markers (*COL2A*, *ACAN*, *COMP*, *SOX-9*), a considerable variability was observed not only across different EV preparations, but also across biological replicates of chondrocytes (Fig. 4b). *COL2A* and *ACAN* were robustly upregulated by CHO-Ti-EVs and PPP-EVs but showed only modest or variable increases with CHO-MSC-EVs (Fig. 4b). In particular, CHO-Ti-EVs induced the highest increase in *COL2A* expression, while CHO-MSC-EVs-P were the only EVs that failed to induce an overexpression of *COL2A*. The expression of *ACAN* was supported by both CHO-Ti-EVs and PPP-EVs, but CHO-MSC-EVs failed to induce its overexpression. *COMP* expression however was specifically supported by CHO-MSC-EVs, and *SOX-9* expression was highly variable and with no distinct pattern observed. Only PPP-EVs supported the expression of all ECM components.

The expression of the *COL10A* hypertrophic marker was also evaluated, but with significant variability between individual replicates of chondrocytes. No difference was observed by the isolation protocol of CHO-MSC-EVs. Only PPP-EVs induced a clearer downregulation of *COL10A*, which may relate to increased *SOX-9* expression in these samples. As *SOX-9* negatively regulates *COL10A* in non-hypertrophic chondrocytes, this pattern suggests a shift toward a more stable chondrogenic phenotype in response to PPP-EVs (Fig. 4a, b). Within other groups of EVs, the expression of both *SOX-9* and *COL10A* was indecisive.

**Fig. 5:**
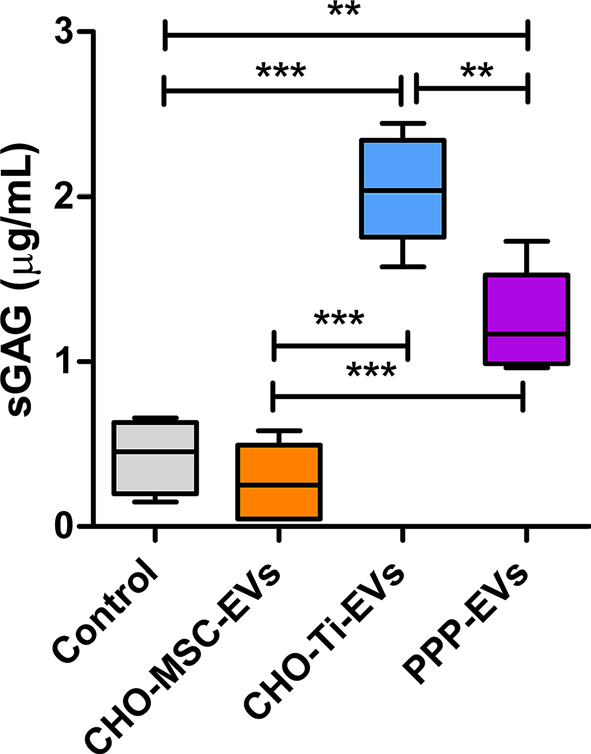
sGAG production in chondrocytes treated with CHO-MSC-EVs-SEC, CHO-Ti-EVs and PPP-EVs, all isolated using SEC. Chondrocytes were treated with EVs for 10 days. Results show 3 biological replicates of OA- chondrocytes. *p < 0.05; **p < 0.01; ***p < 0.001

To complement the gene expression data, sulphated glycosaminoglycan (sGAG) production was assessed as a functional marker of ECM synthesis. After 10 days of treatment, chondrocytes exposed to CHO-Ti-EVs or PPP-EVs showed a significant increase in sGAG production compared to the untreated control (Fig. 5), indicating enhanced matrix deposition. In contrast, sGAG levels in the CHO-MSC-EV-treated group remained comparable to control.

Overall, we demonstrated that CHO-Ti- and PPP-EVs uniquely combine minimal inflammatory signalling with potent activation of ECM synthesis, underscoring their superiority as a regenerative stimulus compared to conventional CHO-MSC-EV preparations.

#### Comparison of the soluble proteins and EV fractions

**Fig. 6:**
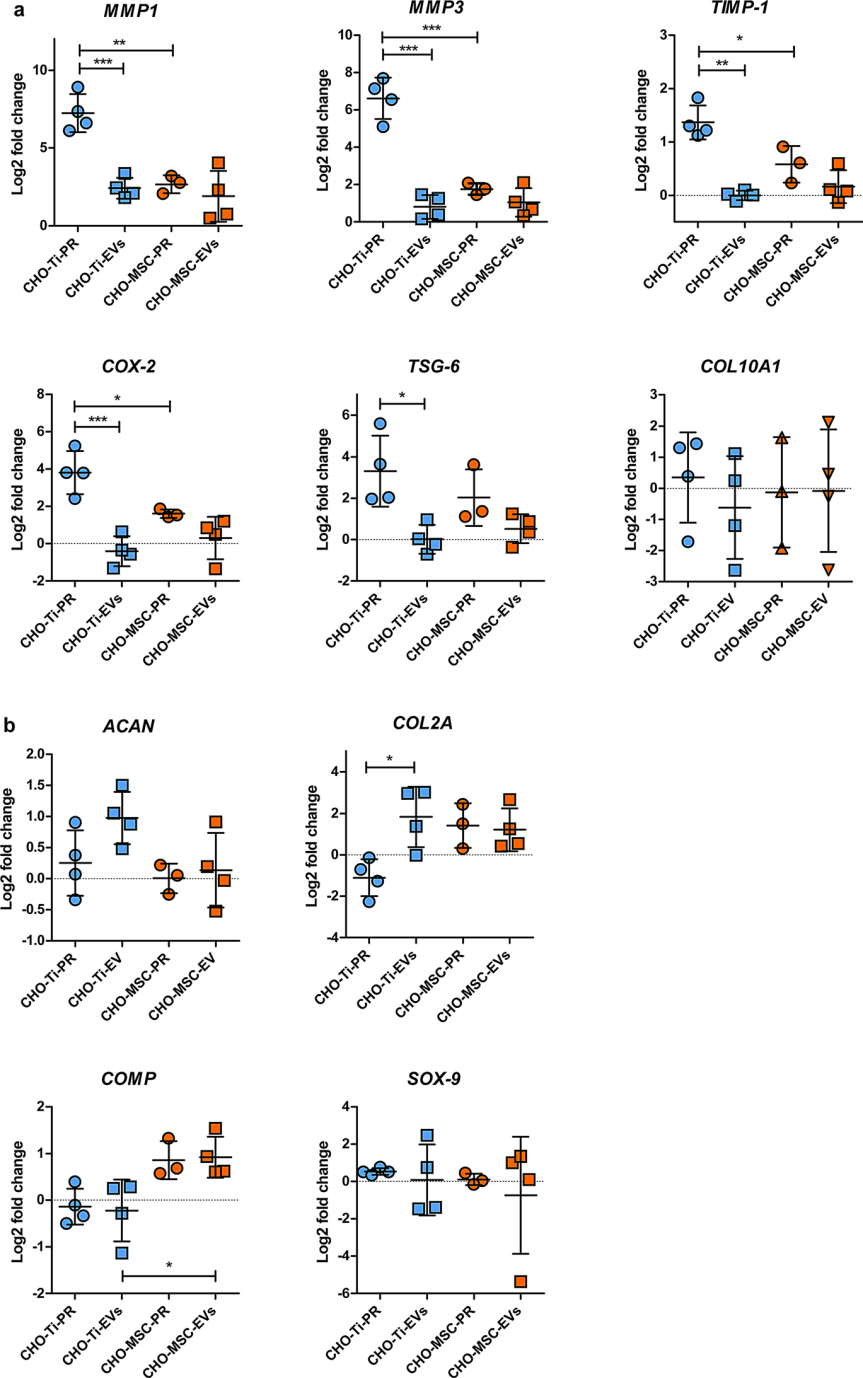
Changes in the expression of pro-inflammatory/degradation (**a**) and chondroprotective (**b**) genes of chondrocytes co-cultivated with the enriched soluble protein (PR) or EVs fraction of CHO-Ti (blue) or CHO-MSCs (orange). Expression is shown as the log2 value of fold change, compared to the expression of the untreated control. Symbols represent individual replicates, the mean ± SD is shown. * *p <* 0.05; ** p < 0.01; *** p < 0.001

Finally, to evaluate whether the beneficial effects of EVs are shared by other components of the secretome, the biological effect of the soluble protein fractions were analysed in comparison with their respective EVs. We observed that CHO-Ti-PR drove a strong inflammatory response, while purification to EVs dramatically reduced inflammatory gene induction. CHO-Ti-PR significantly increased the expression of all pro-inflammatory genes, along with the downregulation of key chondroprotective genes (*COL2A* and *ACAN*, Fig. 6) in comparison with CHO-Ti-EVs. On the other hand, CHO-MSC-EVs and CHO-MSC-PR were more similar, leading to comparable effects, particularly in promoting chondroprotective gene expression. However, their impact varied primarily in the regulation of inflammatory gene expression (*COX-2*, *TIMP-1*,* TSG-6*).

We show that the beneficial effects of CHO-Ti secretome are specifically linked to EVs, which offer a cleaner regenerative stimulus by decoupling the unwanted inflammatory gene response from the desired chondrogenic gene activation.

#### Expression profiling

**Fig. 7:**
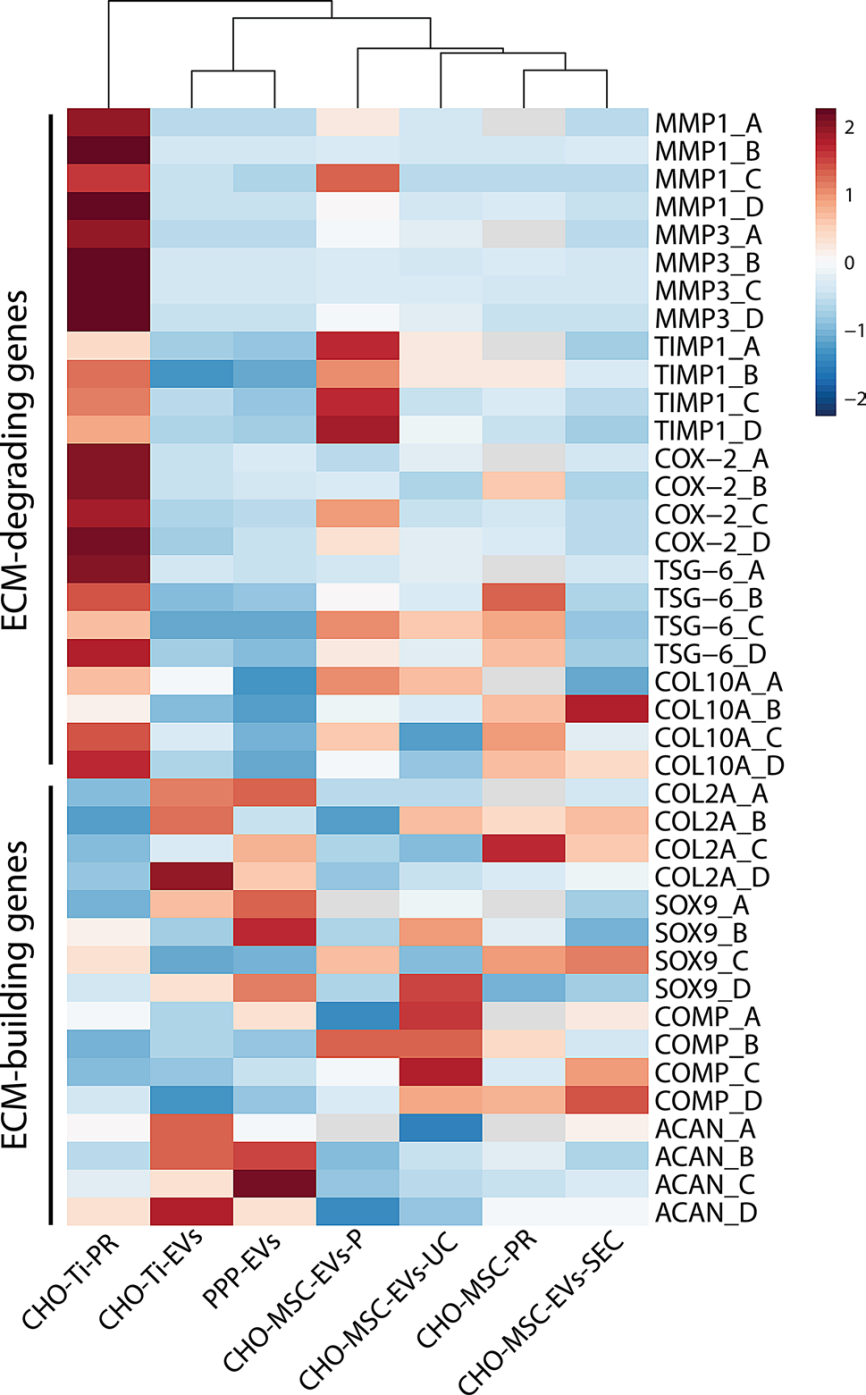
Heatmap of the relative expression of ECM-degrading and ECM-building (chondroprotective) genes of 4 biological replicates (A, B, C, D) of OA chondrocytes co-cultivated with the enriched soluble protein (CHO-Ti-PR and CHO-MSC-PR) or EVs fraction from different EV sources (chorion tissue CHO-Ti, platelet-poor plasma PPP and CHO-MSCs isolated by different methods, such as precipitation CHO-MSC-EVs-P, precipitation + ultracentrifugation CHO-MSC-EVs-UC, size exclusion chromatography CHO-MSC-EVs-SEC). Values are scaled by row and treatment groups are clustered by Euclidean distances

In the gene expression heatmap combining all treatment groups and chondrocyte replicates (Fig. 7), three distinct clusters emerged. The soluble protein fraction of CHO-Ti formed a separate cluster, consistently displaying high expression of inflammatory and degradation genes across all biological replicates. The second cluster comprises CHO-Ti-EVs and PPP-EVs, both exhibiting a very similar profile characterized by low inflammatory gene expression and elevated levels of chondrogenic genes, particularly *ACAN* and *COL2A*. The third cluster includes all CHO-MSC-EVs and CHO-MSC-PR, with CHO-MSC-EVs-P positioned further apart due to its high expression of inflammatory genes. Both CHO-MSC-EVs and CHO-MSC-PR similarly enhanced the expression of chondrogenic genes, specifically *COMP*.

The heatmap also reveals that biological replicates of chondrocytes responded similarly in the expression of inflammatory and ECM-degrading genes (*MMPs*,* TIMP-1*,* COX-2*). However, the expression of chondrogenic genes (*ACAN*,* COL2A*,* COMP*,* SOX9*) varied considerably between the replicates. This variation suggests that the baseline condition of OA chondrocytes, including the existing expression levels of ECM components, may influence their response to different treatments and EV types.

## Discussion

EVs derived from different sources inherently vary in their composition and properties, leading to distinct responses in target cells, likely by influencing different stages of various pathways. Due to these inherent differences, understanding how EVs from various sources influence chondrocytes is crucial for optimizing regenerative therapies.

While MSC- and PL-EVs have already demonstrated potential in chondrocyte and cartilage regeneration (5–9; 12–19), Ti-EVs appear to hold even greater promise but remain largely underexplored. Given the remarkable properties of fetal membranes, such as the amnion and chorion [28, 37], and fetal membrane-derived MSCs [24–27], this study aimed to compare the regenerative effects of CHO-Ti-EVs– a novel and rich source of potent EVs- with CHO-MSC- and PPP-EVs, as already recognized cell-free therapies, by analysing their impact on gene expression changes in OA chondrocytes.

In our study CHO-Ti was the richest source of EVs, followed by PPP. Despite the relatively high content of protein contamination (Fig. 3b, c), EVs isolated from CHO-Ti and PPP exhibited more favourable anti-inflammatory and chondroprotective effects on OA chondrocytes compared to those of CHO-MSC-EVs isolated by three different methods (Fig. 4). PPP-EVs showed the most beneficial effect, exhibiting the lowest expression of ECM-degrading genes, and showing both anti-inflammatory and high chondrogenic potential. Notably, PPP-EVs were the only group to consistently upregulate all key ECM-building genes. CHO-Ti-EVs closely followed in effectiveness, showing an even stronger ability to suppress typical OA-related inflammation markers *COX-2* and *TSG-6*, and to support the expression of key ECM-building genes *COL2A* and *ACAN*, but failing to support *COMP* and *SOX-9*. Importantly, transcriptomic data were supplemented by sGAG quantification, where CHO-Ti-EVs induced the highest sGAG production, followed by PPP-EVs (Fig. 5), indicating enhanced ECM synthesis and functional support for cartilage regeneration. The observed pattern is consistent with ACAN gene expression (Fig. 4b), one of the main proteoglycans in chondrocytes, supporting the functional relevance of our transcriptomic data. Our preliminary findings indicate that both PPP- and CHO-Ti-EVs may exceed the regenerative potential of CHO-MSC-EVs.

Ti-EVs represent a unique and emerging class of EVs with significant potential for both diagnostic and therapeutic applications. Ti-EVs contain diverse subpopulations, each likely playing distinct biological roles, which may lead to different or synergistic effects on recipient cells. While they are particularly valuable from a diagnostic perspective, recent studies have also highlighted their therapeutic potential [38–40]. For instance, Dong et al. [38] demonstrated that Ti-EVs derived from apoptotic adipose tissue can enhance wound healing. Similarly, Wang et al. [39] reported that muscle tissue-derived EVs from exercised mice improved metabolic profiles in obese mice, reduced lipid burden, enhanced liver functionality, and decreased atherosclerotic plaque formation. Additionally, Lou et al. [40] found that neonatal Ti-EVs promote tissue repair and yield a higher number of EVs compared to cell line-derived EVs. However, it is important to emphasize, that healthy tissue is essential to facilitate the regenerative effect, since EVs from pathological or injured tissues may retain disease-associated signals that could potentially induce or exacerbate pathological processes [41].

While our current study used freshly processed tissue for EVs isolation, it remains to be determined whether cryopreservation of chorion tissue prior to EVs isolation affects the yield or therapeutic properties of EVs. Fresh processing is generally considered optimal for maximizing EV yield and functional efficacy, especially in research or clinical-grade EV production. It ensures minimal degradation and preserves the native state of EV cargo [42, 43]. Further studies are needed to evaluate the impact of tissue preservation, as this will influence the feasibility of routine large-scale EV production and clarify the practical implications of tissue availability.

CHO-Ti-EVs were especially rich in the CD146 surface marker, which may correlate with their high anti-inflammatory properties (Fig. 3d, e, Suppl. Fig. S3). The presence of CD146 on EVs originates back to their parental chorionic mesenchymal progenitor cells. Previous studies have shown that CD146 + MSC subpopulations have superior biological functions and therapeutic potential, especially in immunomodulation [44, 45] and CD146 expression is regarded as a quality control standard for MSC products [46]. Furthermore, research indicates that CD146-enriched EVs may offer therapeutic benefits in reducing atherosclerosis [47]. Altogether, these findings highlight that the high CD146 expression on CHO-Ti-EVs may contribute to their anti-inflammatory effects, warranting further confirmation and investigation into their specific mechanisms of action.

Chondrocytes contribute to cartilage regeneration by synthesizing ECM, making this an imperative aspect of cartilage regeneration alongside the immunomodulation [48, 49]. All EV samples, except for the CHO-MSC-EVs-P, induced an overexpression of *COL2A*, the key component of healthy cartilage. The expression of *ACAN* was mainly supported by CHO-Ti- and PPP-EVs, and the expression of *COMP* was specifically supported only by CHO-MSC-EVs, regardless of the isolation technique, and CHO-MSC-derived protein fraction, as well.

COMP is a non-collagenous matrix protein expressed predominantly by chondrocytes. It interacts with collagen type II, helping to maintain the structural integrity of cartilage. It is known that *COMP* and *COL2A1* expression are regulated differently during chondrogenesis in BM-MSCs and articular cartilage [50, 51]. *COMP* expression is primarily stimulated by TGF-β [50, 51], whereas *COL2A1* is tightly controlled by the SOX trio—SOX5, SOX6, and SOX9 [51]. The secretome of fetal membranes, including the chorion, is particularly rich in various growth factors, such as TGF-β [28], but there may be important differences in the amount and isoform variety of secreted TGF-β between CHO-MSCs and CHO-Ti. Studies suggest that CHO-MSCs might secrete higher amounts of TGF-β3, which is the isoform specifically involved in regenerative ECM remodelling [52, 53], than CHO-Ti itself, which exhibits a transient peak in TGF-β3 expression during early gestation but falls at around 9–12 weeks’ gestation [54].

SOX-9 is the master regulator for chondrogenesis and directly activate *COL2A* [55]. Its upregulation often occurs rapidly after exposure to specific stimuli, initiating a cascade of regulatory events. For instance, fibroblast growth factor 2 (FGF2) treatment has been shown to increase SOX-9 mRNA levels within 30 min in primary chondrocytes, indicating a swift response to this growth factor [56]. However, in our study its expression was indecisive by addition of EVs after 24 h co-cultivation, despite upregulation of *COL2A* was achieved with most of the EVs (Fig. 4b). It is possible that the increased expression of *SOX-9* occurred hours before the changes were detected (at 24 h). In a study by Wijenayake et al., 2024 [57], authors followed gene expression changes 6, 9, 12 and 24 h after supplementation by milk-derived EVs and detected the most changes occurred at 12 h post supplementation. Different genes might react within different timelines and 24 h might be too long for the effects to last from a single dose of EVs for certain genes [57]. For this reason, additional studies investigating temporal gene expression changes following EV supplementation would be valuable to reveal optimal timing for observing peak biological effects.

In our study, CHO-MSC-EVs isolated by precipitation (CHO-MSC-EVs-P) induced unfavorable gene expression patterns in chondrocytes (such as increased expression of *MMPs*, *TIMP-1*, and *COX-2;* Figs. 4b and 7), which aligns with existing concerns about this isolation method. Although precipitation is a simple and cost-effective method yielding high quantities of EVs, it is prone to co-precipitation of non-EV contaminants and polymer retention, which can compromise EV purity and introduce unintended bioactivity [58–60]. For example, THP-1-derived macrophages exposed to PEG-isolated EVs from blood plasma showed a clearly reduced viability after 1 and 3 h at 37 °C, compared to EVs isolated by SEC. [58]. In another study only precipitated EVs did not promote cell proliferation [59]. Authors also detected PEG contamination in EV samples and concluded that precipitated EVs likely contained some cytotoxic chemical(s), which inhibited cell growth [59]. In Williams et al., PEG precipitation resulted in a significantly reduced presence of CD81^+^ particles when compared to other isolation methods [61]. In our study, we observed significantly reduced expression of the CD49e surface marker and the EV-specific CD9 in CHO-MSC-EVs-P compared to CHO-MSC-EVs isolated by the other two methods (Fig. 3f), suggesting that the technique may influence either the detection or integrity of EVs surface markers. Additional purification by ultracentrifugation (CHO-MSC-EVs-UC) modestly improved EVs quality, as reflected by higher relative purity (Fig. 3b, c) and improved biological effects. However, CHO-MSC-EVs-SEC demonstrated the most favorable characteristics, including a higher expression of MSC-specific surface markers (Fig. 3f) and more regenerative gene expression responses—such as increased *COL2A* and *ACAN* and reduced expression of ECM-degrading enzymes (Figs. 4 and 7). These findings suggest that the observed bioactivity is strongly influenced by the isolation method rather than the inherent properties of MSC-derived EVs and highlight that precipitation-based methods may be suboptimal for isolating EVs intended for therapeutic or functional studies.

Although UC remains the most popular EVs isolation method, it can co-precipitate protein aggregates or damage particles [62]. This can be overcome by SEC which removes soluble proteins, can produce preparations of high yield, while preserving biophysical and functional properties of the isolated vesicles [59, 63]. SEC is also the gold standard for the isolation of plasma EVs [64] and is becoming increasingly popular for Ti-EVs, either as a final purification step to enhance purity or as a standalone method [65]. Its use depends on tissue type and is often combined with tissue culture.

Factors that are potentially beneficial for regeneration are distributed both within EVs and in the soluble fraction of the CM. In our study, CHO-MSC secretome proteins mimicked MSC-EVs’ effects but induced higher *COX-2* and *TSG-6* expression, unlike soluble proteins of Ti, where the beneficial biological properties seem to be exclusively associated with CHO-Ti-EVs (Fig. 6). Results of studies comparing the effects of CM, EVs and soluble protein fractions are divergent, with some attributing higher immunomodulatory effects to EVs [66], while others report a stronger influence of soluble proteins [67, 68]. The inconsistency may arise from differences in isolation methods, cell sources, and experimental models. Standardized approaches are key to reach consistency.

Despite the promising findings, several limitations of our study should be acknowledged. Although primary OA chondrocytes were used to simulate clinically relevant conditions, in vitro models lack the full physiological complexity of the joint environment, such as 3D matrix architecture, immune cell interactions or mechanical stimulations. Another limitation is that, apart from gene expression analysis, only limited protein-level data were collected (we included sGAG quantification as a functional readout of matrix production). Since our experiment focused on early responses 24 h after a single EVs dose, this timeframe may be insufficient for detectable protein changes in some markers.

It is also important to note that, while CHO-Ti and plasma are abundant and ethically acceptable sources, biological variability among donors may affect EVs composition and reproducibility of effects. Moreover, although we systematically compared EVs isolation methods, the absence of universally accepted protocols for EVs characterization and purity assessment may limit cross-study comparisons. Nevertheless, the strength of our study originates in the direct comparison of EVs from multiple clinically relevant sources under optimized experimental conditions, highlighting unique functional profiles and potential of CHO-Ti-EVs. Our study offers important insight into how EVs source and purification strategies affect their biological activity, supporting further progress in regenerative medicine.

## Conclusion

Ti-EVs have primarily been explored as diagnostic tools, while MSC-EVs are traditionally preferred for therapeutic applications. However, our study challenges this perspective, as CHO-Ti-EVs were associated with more favourable outcomes than CHO-MSC-EVs after a 24 h co-cultivation with OA-chondrocytes. CHO-Ti-EVs, alongside the PPP-EVs, not only provided a significantly higher particle yield but also more effectively suppressed pro-inflammatory gene expression and robustly upregulated ECM–related transcripts, compared to CHO-MSC-EVs. These findings highlight the unique therapeutic potential of CHO-Ti-EVs as an underexplored strategy for chondrocyte support and cartilage regeneration. When tissue availability is not a limiting factor, such as with healthy placental tissues after full-term delivery, CHO-Ti-EVs may represent a promising new class of therapeutic EVs.

## Supplementary Information

Below is the link to the electronic supplementary material.

Supplementary Material 1

## Data Availability

Data supporting the findings of this study are available within the article and the Supplementary Material. Further inquiries can be directed to the corresponding author.

## Abbreviations

CM: Conditioned medium
CHO: Chorion, chorionic
ECM: Extracellular matrix
EVs: Extracellular vesicles
MFI: Mean fluorescence intensity
MSC: Mesenchymal stromal/stem cells
NTA: Nanoparticle tracking analysis
OA: Osteoarthritis
P: Precipitation
PCA: Principal component analysis
PPP: Platelet-poor plasma
PR: Protein
PRP: Platelet-rich plasma
SEC: Size exclusion chromatography
sGAG: Sulphated glycosaminoglycan
Ti: Tissue
UC: Ultracentrifugation
