# Supplementary material for: Chorion tissue- and plasma-derived extracellular vesicles exhibit superior anti-inflammatory and chondroprotective effects
Source: Stem Cell Res Ther. 2025 Aug 5;16:423. doi: 10.1186/s13287-025-04542-9 (PMC12326873; doi:10.1186/s13287-025-04542-9)
Supplement: Supplementary file 1 — Supplementary Material 1 [file 13287_2025_4542_MOESM1_ESM.docx]

**SUPPLEMENTARY MATERIAL**

# **Chorion Tissue- and Plasma-Derived Extracellular Vesicles Exhibit Superior Anti-inflammatory and Chondroprotective Effects**

Livia K. Fecskeova^1^, Jana Matejova^1^, Lucia Slovinska^1^, Jana Bzdilova^1^, Zuzana Kozovská ^2^, Denisa Harvanova^1^

^1^Associated Tissue Bank, Faculty of Medicine, P. J. Safarik University and L. Pasteur University Hospital in Kosice, Tr. SNP 1, 04011 Kosice, Slovakia.

^2^Cancer Research Institute, [Biomedical Research Center SAS](https://www.sav.sk/?lang=en&doc=ins-org-ins&institute_no=198), Dúbravská cesta 9, 845 05 Bratislava, Slovakia

# **Material and methods**

**Supplementary Table S1**. Overview of donor characteristics and eligibility criteria for each tissue source.

|  | **Donor information** | | | | | |
| --- | --- | --- | --- | --- | --- | --- |
| **Source** | **Age** | | **Sex** | | **BMI**  median | **Clinical status ^*^** |
|  | median | range | female | male |  |  |
| **Chorionic tissue**  (n = 4) | 25 | 20 - 28 | 4 | - | ND | healthy, full-term pregnancy |
| **Cartilage tissue**  (n = 4) | 55.5 | 52 - 66 | 4 | 0 | 33.85 | end-stage OA |
| **Whole blood**  (n=4) | 38.5 | 26 - 40 | 2 | 2 | 25.05 | healthy |

NOTE: ND – not defined; ^*^ all donors were randomly selected. **Inclusion criteria:** ***chorionic tissue*** – healthy women with full-term pregnancy undergoing planned caesarean section, negative serology for HBV, HCV, HIV, CMV, HTLV, EBV, toxoplasma and venereal diseases, no pregnancy complications. ***Cartilage tissue*** – patients aged 30 – 80 with radiographically confirmed painful knee OA, eligible for total knee arthroplasty (KL grade III-IV), presence of synovial effusion, able to walk independently or with support. ***Whole blood*** – healthy donors aged 20 – 50 with no history of joint pain (KL grade 0). **Exclusion criteria** for all donor groups: inability to provide informed consent, history of cancer, autoimmune or inflammatory disease, active infection, chronic liver disease, anaemia, clotting disorders, or anticoagulant use, neuromuscular or severe cardiovascular disease, immunosuppression or pregnancy (except for placenta donors), severe OA in joints other than the knee (for OA donors). Rheumatoid arthritis or systemic connective tissue diseases.

## **Immunophenotypic Characterization of CHO-MSCs**

Flow cytometry analysis of cell surface markers expression was performed on CHO-MSCs. Cells were harvested and centrifuged at 300 ×g for 10 min and washed with PBS containing 2% FBS. A minimum of 2× 10^5^ cells were incubated with either fluorescein isothiocyanate (FITC)-, phycoerythrin (PE)- or allophycocyanin (APC)-conjugated antibodies: CD29, CD34, CD45, CD73, CD90 and CD105 for 10 min in the dark. The samples were then washed and centrifuged at 300 ×g for 10 min. The resuspended cell pellets were analyzed with a Becton Dickinson FACSCalibur using CellQuestPro software (Becton Dickinson, Belgium).

## **Differentiation of CHO-MSCs**

To induce differentiation of CHO-MSCs into adipogenic, osteogenic, and chondrogenic lineages, commercially available StemPro® differentiation kits (ThermoFisher) were used. CHO-MSCs were seeded in 24-well plates at 37˚C with 5% CO_2_ at a density of 2 × 10⁴ cells/well for adipogenesis, 1 × 10⁴ cells/well for osteogenesis, and 8 × 10⁴ cells/well for chondrogenesis. When the cells became 70‑80% confluent, the medium was replaced by differentiation induction medium for 2‑3 weeks. The cells were analyzed for osteogenesis, adipogenesis, and chondrogenesis by Alizarin Red staining, Oil Red O staining, and Alcian Blue staining, respectively.

## **RT-qPCR**

PCR conditions were as follows: 50 °C for 2 min for UDG activation, 95 °C for 2 min for initial denaturation, followed by 40 cycles of 95 °C for 20 s, annealing and extension at 60 °C for 1 min including plate reading.

**Supplementary Table S2**. The list of primers used in RT-qPCR and their sequences.

| Gene | Forward primer sequence (5’-3’) | Reverse primer sequence (5’-3’) |
| --- | --- | --- |
| GAPDH | GTCTCCTCTGACTTCAACAGCG | ACCACCCTGTTGCTGTAGCCAA |
| YWHAZ | ACCGTTACTTGGCTGAGGTTGC | CCCAGTCTGATAGGATGTGTTGG |
| MMP1 | ATGAAGCAGCCCAGATGTGGAG | TGGTCCACATCTGCTCTTGGCA |
| MMP3 | CACTCACAGACCTGACTCGGTT | AAGCAGGATCACAGTTGGCTGG |
| TIMP1 | GGAGAGTGTCTGCGGATACTTC | GCAGGTAGTGATGTGCAAGAGTC |
| COX-2 | CGGTGAAACTCTGGCTAGACAG | GCAAACCGTAGATGCTCAGGGA |
| TSG-6 | TCACCTACGCAGAAGCTAAGGC | TCCAACTCTGCCCTTAGCCATC |
| COL2A | CTCCTGGAGCATCTGGAGAC | ACCACGATCACCCTTGACTC |
| SOX-9 | AGGAAGCTCGCGGACCAGTAC | GGTGGTCCTTCTTGTGCTGCAC |
| COMP | GGAGATGCTTGTGACAGCGATC | TGAGTCCTCCTGGGCACTGTTA |
| ACAN | TGCGGGTCAACAGTGCCTATC | CACGATGCCTTTCACCACGAC |
| COL10A1 | CGCTGAACGATACCAAATGCCC | TGGACCAGGAGTACCTTGCTCT |

# **Results**

**Supplementary Figure S1**. Expression of MSC-specific surface markers (CD90, CD73, CD105) and hematopoietic surface markers (CD34, CD45, CD14) of MSCs from chorion (n=5). Data shown as SD±mean.


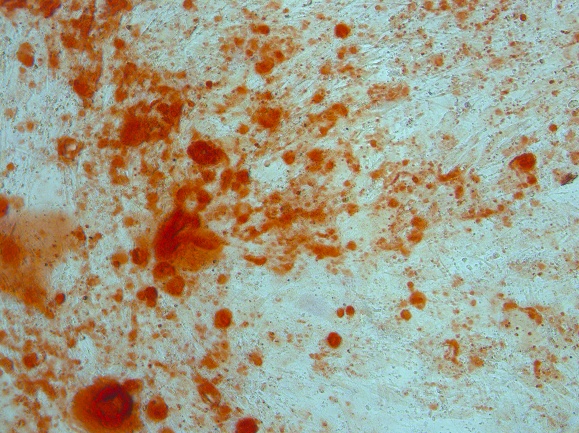

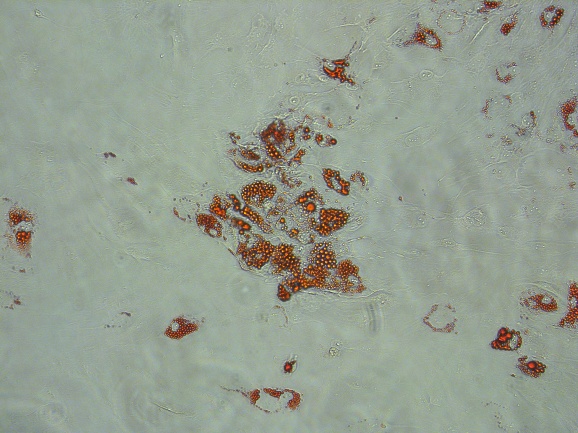

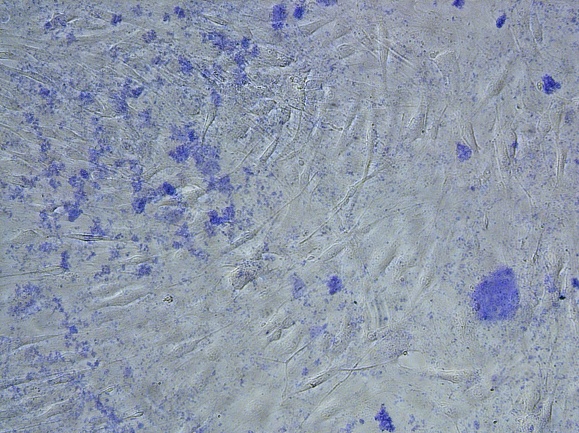


**Oil Red O Alcian Blue Alizarin Red S**

**A**

**B**

**C**

**Supplementary Figure S2.** Adipogenic (A), chondrogenic (B) and osteogenic (C) differentiation of CHO-MSCs. CHO-MSCs were induced to differentiate toward adipogenic, chondrogenic and osteogenic lineages as verified by Oil Red O, Alcian Blue and Alizarin Red S staining, respectively

**Supplementary Figure S3**. Expression of the top 25 EV surface markers on CHO-Ti-EVs (n=4), PPP-EVs (n=4) and CHO-MSC-EVs (n=6). Data shown as SD±mean.

**Supplementary Figure S4**. Expression of the top 15 EV surface markers of CHO-MSC-EVs isolated by precipitation (MSC-EVs-P, n=3), precipitation+ ultracentrifugation (MSC-EVs-UC, n=3) and SEC (MSC-EVs-SEC, n=3). Data shown as SD±mean.
